# A National Doctor Morbidity Survey

**Published:** 1987-11

**Authors:** Huw Griffith

**Affiliations:** Consultant Neurosurgeon, Frenchay Hospital, Bristol


### A National Doctor Morbidity Survey

**Huw Griffith, MA, BM, BCh (Oxon) FRCS FRCP.**
Consultant Neurosurgeon, Frenchay Hospital, Bristol.

Of the many problems which have dogged the Health Service since its inception, one basic one, never properly addressed, has been that of assessing the demand for health-care. Looking at the level of provision over the population as a whole has not been helpful. Attempting to do this has revealed the inadequacy of the machinery for measuring what those who provide health-care are actually engaged in doing. To think that measuring the level of provision would be helpful in deciding what ought to be provided is a fallacy unless we know what the target is, and how far short we fall. Since British medicine falls short in many respects of what is provided in other comparable countries, we do not know whether the differences are important or merely reflect the different financial arrangements for medicine and the motivations which flow from these. Since the difference in medical expenditure per head, for example, between the USA and the UK is approximately three-fold, both these countries cannot be right, given that the measurables (eg. operations performed per unit of population) roughly reflect the financial differences. We certainly need some measure of what levels of health-care provision should be provided, so that we can, when our measurement system can be brought up to scratch, have some idea of what has to be done to achieve that level for the whole of our population. Unless this can be done soon, we shall never conquer the inequalities in the distribution of health-care over our country which the RAWP machinery was meant to try and alleviate.

Twenty-five years ago I spent a year in an African teaching hospital which was surrounded by a high impenetrable wire fence. Only a limited number of people could be admitted each day, so that the unsuccessful ones, surrounded by their squatting relatives, could be seen through the wire in small groups who hoped that they could be admitted the next day, or the one after, or at any rate before the patient's death. Some were unlucky. It was not until my return to the UK that I realised that a similar situation existed here. The only differences were those of degree. The patients, or potential patients, were not so visible. But they are there. Many are not even on waiting lists since their family doctors tend to match up their referral pattern to the facilities available. General practitioners are not usually crusaders for particular brands of health-care.

The persistent existence of waiting lists has given rise to the feeling in the NHS that the demand for health-care is infinite. Those who propagate this view have little evidence for it beyond the existence of waiting lists. By itself, this is not evidence. If we were, for example, able to discover that of a particular operation we needed each year to do exactly the number which were in fact carried out, the existence of a long waiting list would simply be a stimulus to making a temporary extra effort to eliminate the backlog. Another, and much less expensive, way of dealing with the waiting list would be to make it more difficult to put patients on it. This has happened with ENT and tonsilectomy. Due to a change of view among paediatricians and others, both the total numbers carried out and the waiting list for the operation have been brought down. Please note that the ENT surgeons did not spearhead this initiative. Those who duty it is to stagger under the burden of a waiting list rarely have the time or facilities to search for another way around the problem. It is made painfully obvious that an increase in facilities is not to be forthcoming.

A way out of the difficulty of not knowing how much health-care should be provided, even for a modest segment of the NHS, would be well worth having. I believe that this information could be obtained rapidly and relatively cheaply from doctors in respect of their own illnesses, and particularly for operations.

My reasons for believing this are as follows. Since 1979, when we introduced the operation of microdiscectomy for lumbar disc prolapse to this country, we have in Bristol carried out approximately 500. Of these 18 were in doctors. The ratio of doctors to patients (hospital and general practice) in this country is 1:940 (Health Service Data Book). So we should have operated upon approximately half a doctor. We operated, in fact, on 36 times as many. After correcting for the disc operations carried out by the Orthopaedic surgeons in this area, we arrive at a figure of 20–25 times as many operations in doctors as in the general population. Doctors are not known to be selectively vulnerable to disc disease, although they are known to be vulnerable to suicide, alcoholism, and divorce. Our figure for the doctors' disc surgery rate approximates to the 200,000 annually in the 225,000,000 population of the USA. This is approximately one per 1,000 patients at risk per annum. In addition, we have looked at the rate of disc operations in a town and in a country practice with good access to the Neurosurgeons. Both practices refer patients for operation at approximately this rate. Another single-handed general practitioner locally whose has been made aware of the affect of this operation now refers patients at this rate. The rate is likely to be much in excess of that for patients with this disabling condition dealt with by the Health Service generally. It is also very much greater than the rate for privately insured patients (1:17,000 for BUPA subscribers). It is probably greater than for doctors in other parts of the country.

The message which emerges is that doctors (locally) are both aware of the benefits of this operation and also have access to it. In fact, doctors, and perhaps their close relatives, form the only segment of our population which has almost unrestricted access to the facilities of the Health Service. A very large segment of doctors does not carry private medical insurance for they know that they can obtain the treatment they require on the NHS.

Consequently, it becomes important to discover what health-care doctors have obtained. Naturally, some items are easier to measure than others and it would be wise to concentrate on these. Operations in particular are relatively easy to record. In this connection, it is important to understand that the implied contract between a doctor as patient and the surgeon is likely to differ from that of an "ordinary" patient. For instance, patients are likely to be less critical of surgeons and surgery than doctors are. Doctors are less likely to submit themselves to operations without crisp and substantial reasons. Similarly, the surgeon is likely to be more aware of the possibility of professional disgrace were an operation on a doctor to go wrong. It is unlikely that doctors are over-operated upon.

If we were to obtain reliable figures for the operations conducted on the only national sample (approximately 60,000) who have unrestricted access to the Health Service, we would still have to interpret these carefully. For instance, doctors are in social class 1 and their morbidity pattern may differ from that of other social classes. In order to become a doctor, it is necessary to endure a physically and mentally demanding long medical course. Those who emerge successfully have already been screened. Nevertheless, it should be possible to make intelligent guesses about how doctors differ from other segments of the population with similar educational background and lifestyles. It is recognised that the "middle class" are adept at obtaining the health care they want, but no other class has the access which doctors have.

How is this information to be obtained? Unfortunately, occupation is not adequately coded in the national morbidity statistics (it should be!). Equally, the nationally available figures are so non-specific as to be largely useless as far as operations are concerned. I believe that a postal ballot should be attempted—a reply-paid postcard asking all doctors what episodes of health care and operations they have had in the last five years. When these have been analysed, we shall be able to see how representative they may be. Doctors must maintain a registered address for GMC purposes and are likely to be more contactable by post than the bulk of the population. Provided the approach is adroitly made, there should be a good expectation of co-operation and reply. Further information to amplify and correct could be obtained using the telephone.

Such an effort would take a modest sum of money only for postage and collecting the replies. It need to be taken only over one or two years, for logistic convenience and only if it were providing further useful and relevant information would it be necessary to persist. Lest it be thought that knowing what doctors are having done for themselves will inevitably lead to a cry for increased expenditure on health-care, consider what the consequences would be, for instance, were female doctors to have hysterectomies at a much lower rate than social class 5 females.

Were such a survey to point out gross discrepancies between the figures for certain operations in doctors and for the population as a whole, it would be important to mount subsidiary enquires to find out why. We are already embarked on such a study here in Bristol for disc disease where we have the interest and the diagnostic facilities to mount it. We anticipate that the discovery of the true Health Service incidence of disc prolapse (that which has exceeded the management facility of the general practitioner) requiring further diagnosis and treatment, will give us data which, for the first time, will enable us to plan nationally for dealing with this painful and disabling and expensive disease. General surgeons to whom I have talked believe that the provision for the disease treated by their specialty is probably enough, given a modicum of organisation, to address its task successfully i.e. to keep pace with demand. To continue to labour away depressed by a waiting list which might easily be abolished, given some ingenuity, would be replaced, could we but show it, by an uplifting conviction that a realistic goal can be achieved. In addition, knowing at what rate doctors are being realistically treated will provide a politically irresistible lobby for more facilities if these are in fact required. Politicians, for their part, could be shown that instead of always calling blindly for more, doctors are now able to present them with realistic and achievable targets, with a price-tag attached. If the discrepancy between the target and the facilities potentially available is demonstrably too great, a search for realistic alternative treatment methods will be stimulated. For instance we are already developing an outpatient aspirator suitable for approximately half the prolapsed discs—probably not quite as efficient as an operation but quick and relatively non traumatic. Similarly I have successfully devised a technique for operating on lumbar disc prolapses as day cases, much reducing the cost of the operation.

Based on measured realities I believe that an attempt to introduce a national plan for the Health Service must come rapidly and urgently. Once we begin to measure it, we shall see that the demand for health-care is far from being infinite. This must give those who work in the Health Service a great stimulus, sorely needed, to start solving the real problems of health in our country.

Presidential Address to the Bristol Medico-Chirurgical Society, October 1986.

